# Sinomenine Inhibits Vasculogenic Mimicry and Migration of Breast Cancer Side Population Cells via Regulating miR-340-5p/SIAH2 Axis

**DOI:** 10.1155/2022/4914005

**Published:** 2022-03-09

**Authors:** Lingqin Song, Liqiong Tang, Dalin Lu, Min Hu, Chengcheng Liu, Haifeng Zhang, Yang Zhao, Di Liu, Shuqun Zhang

**Affiliations:** ^1^Department of Oncology, The Second Affiliated Hospital, Xi'an Jiaotong University, Xi'an, 710004 Shaanxi, China; ^2^Department of Epidemiology, School of Medicine, Jinan University, Guangzhou, 510632 Guangdong, China; ^3^Department of Applied Chemistry, School of Science, Xi'an Jiaotong University, Xi'an, 710049 Shaanxi, China; ^4^Department of Pathogenic Microbiology & Immunology, Xi'an Jiaotong University Health Science Center, Xi'an, 710061 Shaanxi, China; ^5^Department of Pathology, Xi'an Jiaotong University Health Science Center, Xi'an, 710061 Shaanxi, China

## Abstract

Hypoxia and its induced vasculogenic mimicry (VM) formation, which both closely related with stem-like side population (SP) cells, are the main culprits leading to tumor invasion and metastasis. Sinomenine exhibits excellent anticancer activity in breast cancer, but whether and how it affects hypoxia-triggered VM formation in breast cancer SP cells remains unclear. In this study, breast cancer SP cells were sorted from MDA-MB-231 cells and cultured with sinomenine under hypoxic conditions. Sinomenine obviously repressed the migration and VM formation of breast cancer SP cells. Through downregulating SIAH2 and HIF-1*α*, sinomenine can inhibit epithelial-mesenchymal transition process of breast cancer SP cells. SIAH2 was identified as a target of miR-340-5p and was downregulated by it, and sinomenine can upregulate miR-340-5p. Hypoxia-induced downregulation of miR-340-5p and activation of SIAH2/HIF-1*α* pathway can be both counteracted by the sinomenine. Moreover, miR-340-5p inhibition and SIAH2 overexpression can partly counteract the anticancer effects of sinomenine. Taken together, sinomenine inhibits hypoxia-caused VM formation and metastasis of breast cancer SP cells by regulating the miR-340-5p/SIAH2 axis.

## 1. Introduction

As the third most prevalent malignant tumor worldwide, breast cancer caused millions of illnesses and hundreds of thousands of deaths every year globally [1]. Despite great advances in the diagnosis and management of breast cancer over the past few decades, some patients with high-metastatic types still exhibit poor prognosis and poor overall survival. Thus, it is of great importance to develop more effective agents for advanced breast cancer patients.

The survival, growth, invasion, and metastasis of malignant tumors need to obtain nutrients from the host environment via neovascularization [2]. Vasculogenic mimicry (VM), which is considered a microvascular channel-like structure formed by nonendothelial cells [2], has been generally recognized as a new pattern of neovascularization which closely associated with the tumor tumorigenesis, aggressiveness, metastasis, drug resistance, and poor prognosis in multiple aggressive malignancies [3]. Multiple factors, such as hypoxia inducible factor (HIF)-1*α*, vascular endothelial (VE)-cadherin, epithelial-mesenchymal transition (EMT), matrix metalloproteinases (MMPs), and several microRNAs (miRs) [2], have been reported to be involved in the formation of VM and regulate the malignant behavior of breast cancer cells [3]. Therapies targeting to VM or these factors that can regulate VM formation may be a new hope for advanced breast cancer patients.

Cells with stem characteristics found in different types of cancer have been confirmed to be actively involved in VM formation [2–4]. Cancer stem cells (CSCs) have self-renewing and multidirectional differentiation abilities [5] and exhibit increased invasive capacity and in vivo tumorigenicity [6]. Side population (SP) cells are a type of cells with CSCs characteristics, which play key roles in tumorigenesis, resistance, and recurrence [6, 7]. The breast cancer SP cells were found to be closely related to the malignant behavior of breast cancer cells and the resistance to chemotherapy and radiotherapy [6, 7]. Therefore, it is of great significance to develop therapies that inhibit the formation of VMs in breast cancer SP cells.

Sinomenine is a kind of isoquinoline alkaloids extracted from the plant *Sinomenium acutum* [8, 9]. It has been reported that sinomenine and its derivatives have excellent anticancer activity in many kinds of cancer cells [10]. Our previous researches have shown that sinomenine can inhibit the invasion of breast cancer cells and breast cancer SP cells [9, 11], reduce the metastasis and growth of breast cancer cells, and improve the survival rate of breast cancer-bearing mice [8]. Besides, through inhibition of neovascularization, sinomenine can suppress the metastasis of human osteosarcoma cells [12]. Appropriate dose of sinomenine hydrochloride can inhibit EMT and CSC properties of breast cancer cells [13], and induce vasculature normalization to repress the progression of breast cancer [14]. However, whether and how sinomenine regulate the VM formation of breast cancer CSC-like SP cells are largely unknown.

Since hypoxia is the main culprit causing tumor invasion and metastasis [15], and the breast cancer CSC populations has a higher proportion in MDA-MB-231 cells than other breast cancer cell lines [16], this study explored the effects of sinomenine on hypoxia-triggered VM formation and metastasis of breast cancer SP cells sorted from MDA-MB-231 cells. With the administration of sinomenine, hypoxia-induced VM formation and metastasis of breast cancer SP cells were reduced, accompanied by the upregulation of miR-340-5p and downregulation of SIAH2. Thus, sinomenine and miR-340-5p/SIAH2 axis may the promising target strategies for the management of breast cancer.

## 2. Materials and Methods

### 2.1. Cell Culture and Side Population Sorting

Breast cancer MDA-MB-231 cells were obtained from the Cell Bank of the Chinese Academy of Science (Beijing, China) in March 2019. Cell lines were authenticated with short tandem repeat (STR) DNA profiling by the provider and were negative for mycoplasma. In a 5% CO_2_ environment at 37°C, cells were cultivated in RPMI-1640 medium (Sigma, St. Louis, MO, USA) adding with 10% fetal bovine serum (FBS; Gibco, Carlsbad, CA, USA). Side population (SP) cells were prepared from MDA-MB-231 cells as the previously described methods [11]. Briefly, the well-grown cells were collected and then made into a single-cell suspension (1 × 10^6^ cells/mL). Cells were then incubated with 5 *μ*g/mL of Hoechst 33342 (Sigma) with or without 5 *μ*mol/L of verapamil (Sigma), a calcium membrane channel blocker, in the dark for 90 min at 37°C, with interval mixing every 10 min. Then the reaction was stopped with ice bath, the resulting cell suspension was then centrifuged at 1500 rpm for 10 min at 4°C to pellet the cells. After resuspension, 1 *μ*g/mL propidium iodide (Sigma) was added to identify and exclude dead cells. Finally, cells were sorted by using FACS Aria flow cytometry.

### 2.2. Cell Transfection

After temporal synchronization, the prepared cells were, respectively, transfected with miR-340-5p mimics, miR-340-5p inhibitor, or the negative control miR-NC, which were all obtained from GenePharma (Shanghai, China). The recombinant plasmids pcDNA3.1-SIAH2 was also synthesized by GenePharma. Transfections were conducted in the well-grown cells by using Lipofectamine™ 3000 (Invitrogen, Carlsbad, CA, USA), and confirmed by RT-qPCR.

### 2.3. Sinomenine Treatment

After dissolved with DMSO, sinomenine (Selleck, Shanghai, China) was diluted into different doses in the culture medium. The well-grown cells were inoculated in 24-well plates to grow overnight, and then cultured with 0, 0.25, 0.75, and 1 mM of sinomenine under normoxic conditions (5% CO_2_ and 95% air) or hypoxia ambience (1% O_2_, 94% N_2_, and 5% CO_2_) for 24 h. Cell viability was analyzed by CCK-8 assay as previous described, and 0.75 mM of sinomenine was chosen to conduct further study.

### 2.4. Wound Healing Assay

To test the migration ability of SP cells, the cells were inoculated in 6-well plates and incubated overnight to reach approximately 80% confluence. A scratch wound was created by a sterile pipette tip, followed by culturing with 0.75 mM of sinomenine under normoxic or hypoxia atmosphere. Cell migration was observed and imaged at 0 h and 24 h with an inverted microscopy (Olympus IX70; Olympus Corporation, Osaka, Japan).

### 2.5. Transwell Migration Assay

Cell migration was evaluated by transwell inserts (Corning, Inc., Corning, NY, USA). The well-grown cells were collected and prepared into cell suspension with serum-free medium. 200 *μ*L of cell suspension which containing 5 × 10^4^ SP cells were added in the upper chamber and incubated with 0.75 mM of sinomenine. 600 *μ*L of serum-containing conventional medium was filled in the lower chamber. After 24 h of culture under normoxia or hypoxia, the migrated cells were fixed with paraformaldehyde and stained with crystal violet. At least five randomly selected fields of view were counted and photographed under a light microscope.

### 2.6. Matrigel-Based Tube Formation Assay

After precoated with matrigel (Corning), 24-well cell plates were used to perform the tube formation assay to evaluate the VM-forming capacity in SP cells. The well-grown cells were resuspended in 100 *μ*L culture medium containing 0.75 mM of sinomenine and separately added onto the matrigel-coated wells. Cells were then incubated at 37°C under normoxia or hypoxia until the vascular structures were observed. Photographs of VM formation were captured under a microscope, and the number of tubular structures was counted.

### 2.7. Bioinformatics Prediction and Dual-Luciferase Reporter Gene Assay

According to the results of TargetScan, starBase, miRcode, etc., miR-340-5p was seen to harbor the binding site with SIAH2. This target relationship was verified by a dual-luciferase reporter gene assay kit (Promega, Madison, WI, USA). The mutant (mut) or wild-type (wt) luciferase reporter constructs of SIAH2 that absence or presence miR-340-5p binding sites were respectively co-transfected into HEK293T cells with miR-340-5p mimics or miR-NC via Lipofectamine™ 3000. The luciferase activities were measured after 48 h of transfection.

### 2.8. Quantitative Real-Time Polymerase Chain Reaction (qRT-PCR)

According to the manufacturer's protocols, TRIzol Reagent (Invitrogen) was used to extract total RNA from cells in each group. The template cDNA was synthesized by using a reverse transcription kit (Applied Biosystems, Foster City, CA, USA). RT-PCR was conducted with a SYBR Premix Ex Taq™ II Kit (Takara, Dalian, China) on an ABI 7500 PCR System (Applied Biosystems). The relative expression of the target genes was normalized with U6 or *β*-actin, with the calculation by the 2^−*ΔΔ*CT^ method.

### 2.9. Western Blot

Cells were treated with RIPA buffer (Beyotime, Shanghai, China) to extract total proteins. After quantitation using a BCA kit (Bio-Rad, Hercules, CA, USA) and separation by SDS-PAGE, protein samples were electroblotted onto nitrocellulose membranes (Millipore, Billerica, MA, USA). After sealed with 5% fat-free milk for 1 h, the membranes were incubated overnight with the specific primary antibodies (Abcam, Cambridge, UK) against SIAH2, HIF-1*α*, VE-cadherin, MMP-9, EphA2, E-cadherin, N-cadherin, *β*-actin, and Snail at 4°C, followed by 1 h of incubation with the HRP-linked secondary antibodies at room temperature. The protein bands were finally visualized and analyzed by ECL detection system and ImageJ software.

### 2.10. Statistical Analysis

All experiments in this study are repeated at least three times, and the result data were analyzed and processed in SPSS 22.0 and GraphPad Prism 8.0 software and expressed as the mean ± standard deviation. Student's *t* test or one-way analysis of variance was used to compare the differences between two or more groups, and *P* < 0.05 was considered significant difference.

## 3. Results

### 3.1. Sinomenine Represses Hypoxia-Induced Vasculogenic Mimicry Formation and Metastasis of Breast Cancer SP Cells

**Figure 1:**
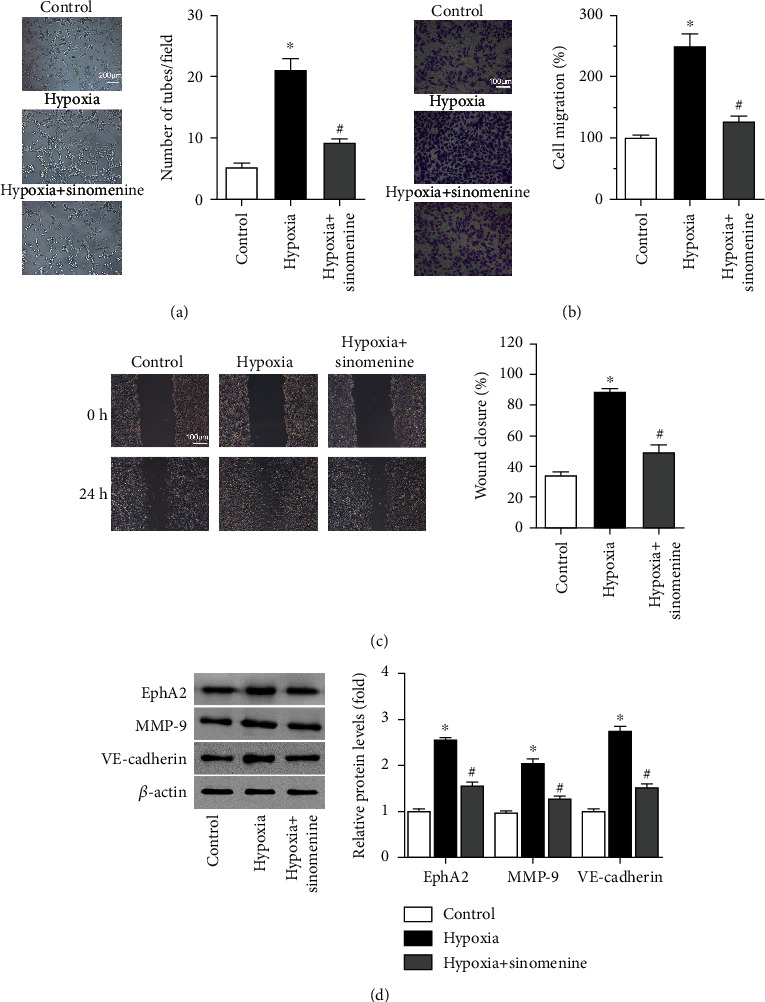
The effect of sinomenine on vasculogenic mimicry formation and migration of breast cancer SP cells. SP cells derived from MDA-MB-231 cells were incubated with 0.75 mM of sinomenine under normoxic or hypoxia for 24 h. (a) Vasculogenic mimicry formation was evaluated by tube formation assay. (b) Cell migration was determined by the transwell assay. (c) Cell migration was observed by wound healing assay and imaged at 0 h and 24 h. (d) The protein expression of EphA2, MMP-9, and VE-cadherin was determined by western blot, and showed in the bar graph. Data are shown as the mean ± standard deviation from 3 independent experiments. ^∗^*P* < 0.05 compared to the control group. #*P* < 0.05 compared to the hypoxia group.

Since low dose of sinomenine had no significant effect on cell viability of breast cancer SP cells (Figure S1), but can effectively reduce their invasion [11], 0.75 mM of sinomenine was applied to breast cancer SP cells to investigate its effects on hypoxia-stimulated VM formation and metastasis. As a result, with the more VM formation induced by hypoxia (Figure 1(a)), SP cells exhibited increased cell migration (Figures 1(b) and 1(c)), as compared to the control cells under normoxia. However, sinomenine markedly repressed the formation of VM (Figure 1(a)) and hindered the migration and wound healing of breast cancer SP cells (Figures 1(b) and 1(c)). Simultaneously, the expression of VM-related proteins, including EphA2, VE-cadherin, and MMP-9 was also notably elevated by hypoxia, but decreased by sinomenine (Figure 1(d)). These data indicate that sinomenine can obstruct hypoxia-induced VM formation of breast cancer SP cells, thereby repressing their metastasis.

### 3.2. Sinomenine Downregulates SIAH2 to Inhibit Epithelial-Mesenchymal Transition of Breast Cancer SP Cells

**Figure 2:**
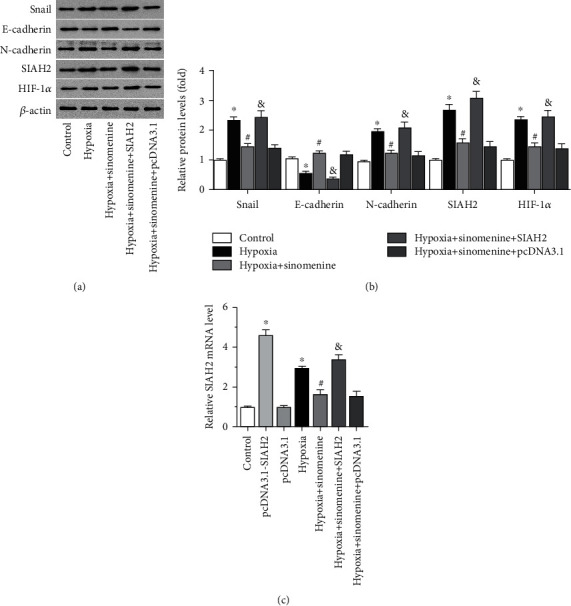
Sinomenine downregulates SIAH2 to inhibit epithelial-mesenchymal transition of breast cancer SP cells. Breast cancer SP cells transfected with pcDNA3.1-SIAH2 or pcDNA3.1 were incubated with 0.75 mM of sinomenine under normoxic or hypoxia for 24 h. (a) The protein levels of Snail, N-cadherin, E-cadherin, SIAH2, and HIF-1*α* were observed by western blot. (b)The relative protein expression levels are shown in the bar graph. (c) The mRNA level of SIAH2 was tested by RT-PCR. Data are represented as the mean ± standard deviation from 3 independent experiments. ^∗^*P* < 0.05 compared to the control group. #*P* < 0.05 compared to the hypoxia group. &*P* < 0.05 compared to the hypoxia + sinomenine group.

According to previous studies, epithelial-mesenchymal transition (EMT) is closely related to the VM formation, migration, and invasion of tumor cells [2–4]. As seen in Figure 2, hypoxia promoted the EMT process of breast cancer SP cells, as evidenced by the reduced expression of E-cadherin and the increased expression of Snail and N-cadherin (Figures 2(a) and 2(b)). However, sinomenine obviously inhibited hypoxia-induced EMT through upregulating E-cadherin and downregulating Snail and N-cadherin (Figures 2(a) and 2(b)). HIF-1*α* plays crucial roles in hypoxia-caused malignant transformation of tumor cells, as well as EMT and VM formation [17–20]. SIAH2, which is closely related to the progression of breast cancer [21], has been reported to affect the stability of HIF-1*α* [22–24]. Herein, hypoxia-induced upregulation of SIAH2, along with increased expression of HIF-1*α*, which were all neutralized by sinomenine (Figures 2(a)–2(c)). Besides, overexpression of SIAH2 obviously alleviated the anti-EMT effects of sinomenine, along with the upregulation of HIF-1*α* (Figures 2(a)–2(c)). These results suggest that sinomenine can repress the EMT process of breast cancer SP cells by inhibiting the SIAH2/HIF-1*α* axis.

### 3.3. Sinomenine Upregulates miR-340-5p to Repress the SIAH2/HIF-1*α* axis in Breast Cancer SP Cells

**Figure 3:**
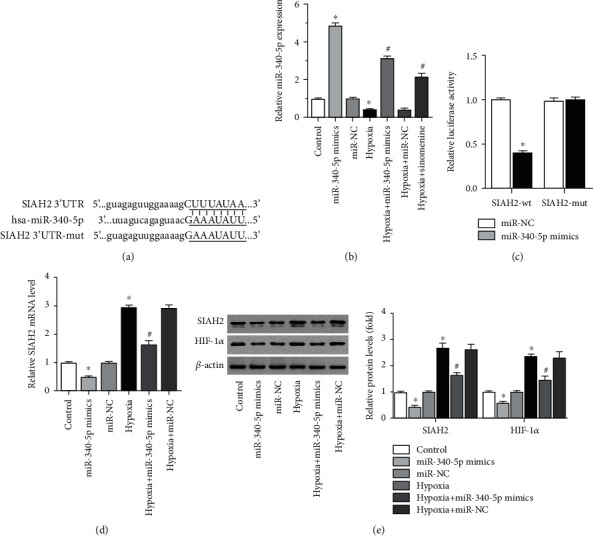
Sinomenine upregulates miR-340-5p to repress SIAH2/HIF-1*α* axis in breast cancer SP cells. (a) Bioinformatics analysis showed the predicted binding sites between miR-340-5p and SIAH2. (b) Breast cancer SP cells were transfected with miR-NC or miR-340-5p mimics, and cultured with 0.75 mM of sinomenine under normoxic or hypoxia, the level of miR-340-5p was assessed by RT-PCR. (c) HEK293T cells were cotransfected with miR-340-5p mimics or miR-NC and SIAH2-wt or SIAH2-mut, the relative luciferase activity was assessed by the dual-luciferase reporter gene assay. (d) The mRNA level of SIAH2 was evaluated by RT-PCR. (e) The protein levels of SIAH2 and HIF-1*α* was assessed by western blot. Data are represented as the mean ± standard deviation from 3 independent experiments. ^∗^*P* < 0.05 compared to the control group. #*P* < 0.05 compared to the hypoxia group.

Given that multiple miRNAs are involved in cell carcinogenesis through the regulation of multiple genes, miRNA-related bioinformatics analysis was performed to investigate the possible regulatory mechanism of the SIAH2/HIF-1*α* axis. As shown in Figure 3 (a), miR-340-5p, a miRNA closely associated with breast cancer invasion and migration of breast cancer [25], was found to harbor a putative binding site with SIAH2 (Figure 3(a)). Transfection of miR-340-5p mimics notably improved the expression of miR-340-5p and neutralized hypoxia-induced downregulation of miR-340-5p (Figure 3(b)). Moreover, miR-340-5p mimics significantly reduced the luciferase activity of the wild-type SIAH2 plasmid while had no effect on the mutated one (Figure 3(c)). The expression of SIAH2 and HIF-1*α* was also downregulated by miR-340-5p mimics (Figure 3(d) and 3(e)). Unlike with the upregulation of SIAH2 and HIF-1*α* induced by hypoxia (Figure 3(e)), miR-340-5p was decreased by hypoxia in breast cancer SP cells (Figure 3(b)). Moreover, transfection of miR-340-5p mimics can significantly inhibited the expression of SIAH2 and HIF-1*α* in breast cancer SP cells exposed to hypoxia (Figure 3(e)). In addition, sinomenine notably elevated the levels of miR-340-5p in breast cancer SP cells exposed to hypoxia (Figure 3(b)) and reduced the expression of SIAH2 and HIF-1*α* (Figures 2(a) and 2(b)). These results indicate that hypoxia can downregulate miR-340-5p to activate the SIAH2/HIF-1*α* pathway, while sinomenine can upregulate miR-340-5p to inhibit the SIAH2/HIF-1*α* axis in breast cancer SP cells.

### 3.4. SIAH2 Overexpression or miR-340-5p Inhibition Counteracts the Anticancer Effects of Sinomenine for Breast Cancer SP Cells

**Figure 4:**
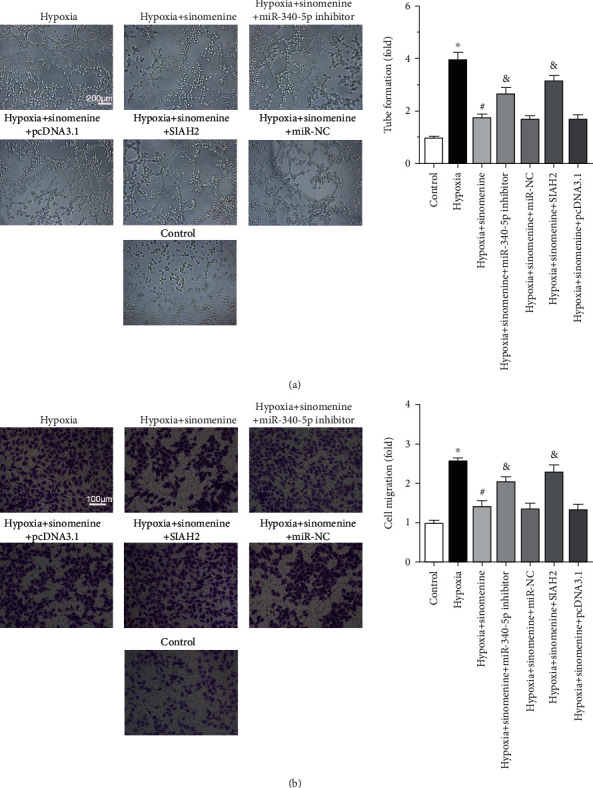
Sinomenine restrains malignant phenotype of breast cancer SP cells via the modulation of miR-340-5p/SIAH2 axis. Breast cancer SP cells transfected with miR-340-5p inhibitor or pcDNA3.1-SIAH2 were incubated with 0.75 mM of sinomenine under normoxia or hypoxia for 24 h. (a) Changes of vasculogenic mimicry formation were evaluated by tube formation assay and showed in the bar graph. (b) Changes of cell migration were determined by transwell assay and showed in the bar graph. Data are expressed as the mean ± standard deviation from 3 independent experiments. ^∗^*P* < 0.05 compared to the control group. #*P* < 0.05 compared to the hypoxia group. & *P* < 0.05 compared to the hypoxia + sinomenine group.

Since hypoxia downregulates miR-340-5p to activate the SIAH2/HIF-1*α* pathway, while sinomenine can upregulate miR-340-5p to inhibit SIAH2, it is suspected that sinomenine can exert anticancer effect through regulating the miR-340-5p/SIAH2 axis. As expected, the inhibition of sinomenine on hypoxia-triggered VM formation and migration of breast cancer SP cells were effectively neutralized by miR-340-5p inhibitor or SIAH2 overexpression (Figures 4(a) and 4(b)). These results indicate that sinomenine can inhibit vasculogenic mimicry and metastasis of breast cancer SP cells by regulating the miR-340-5p/SIAH2 axis.

## 4. Discussion

**Figure 5:**
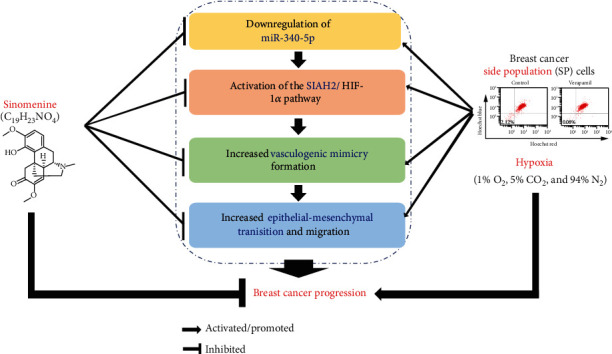
Sinomenine inhibits hypoxia-induced angiogenic mimicry and migration of breast cancer side population cells by modulating the miR-340-5p/SIAH2 axis.

As the main culprit leading to cancer progression, hypoxia and its induced VM formation play important roles in tumor invasion and metastasis [2, 26]. CSCs are believed to be involved in the formation of the VM [2–4], and CSC-like SP cells are also closely related to the malignant behavior of breast cancer cells [5–7]. Sinomenine and its derivatives exhibit excellent anticancer activity in breast cancer [8, 9], but whether and how it affects the VM formation of breast cancer SP cells remains unclear. This study revealed that sinomenine can repress the VM formation and metastasis of breast cancer SP cells by modulating the miR-340-5p/SIAH2 axis (Figure 5).

As a class of isoquinoline alkaloids, sinomenine and its derivatives have many pharmacological activities, such as anticancer and immunomodulatory activities [8–10]. Our previous study indicated that low dose of sinomenine has little cytotoxicity on breast cancer cells and their SP cells [9, 11], but effectively inhibits the invasion and migration of breast cancer cells [9], as well as suppresses the invasive ability of breast cancer SP cells [11]. Sinomenine hydrochloride reportedly represses breast cancer metastasis through inhibiting inflammation-related EMT and cancer stemness [13], which were both related to VM formation [2]. In this study, administration of sinomenine can effectively repress hypoxia-caused VM formation and migration of breast cancer SP cells, with the downregulation of VE-cadherin, MMP-9, and EphA2, which are all closely related to VM formation and metastasis of cancer cells. These findings are similar with our previous research indicated that sinomenine can repress the growth, invasion, and metastasis of breast cancer cells [8, 9]. By inhibiting neovascularization, sinomenine can also suppress the metastasis of human osteosarcoma cells [12]. Sinomenine hydrochloride has also been reported to inhibit breast cancer progression by inducing vasculature normalization [14]. Therefore, sinomenine can obstruct the progression of breast cancer by inhibiting the VM formation of breast cancer SP cells, thereby repressing their metastasis. However, the antiangiogenic effect of sinomenine needs to be validated in more cell lines and model animals in the future.

It is reported that SP cells from breast cancer MCF-7 and MDA-MB-231 cells both showed antiapoptotic and proEMT properties [16, 27]. The increase in EMT phenotype is considered as the important factors in both VM formation and tumor metastasis [2–4]. This study revealed that the expression of EMT-associated proteins, such as Snail and N-cadherin, was notably elevated by hypoxia but decreased by sinomenine. Through reversing EMT process, sinomenine hydrochloride is capable of inhibiting the metastasis of human glioblastoma cells [28]. Sinomenine hydrochloride also obstructed breast cancer metastasis via attenuating EMT and stemness of breast cancer cells [13]. Therefore, inhibiting the EMT of SP cells is one of the mechanisms by which sinomenine restrains the VM formation and metastasis of breast cancer cells.

HIF-1*α* plays crucial roles in hypoxia-induced malignant transformation of many tumor cells [17, 18], including the EMT phenotype and the formation of VM [20, 29]. SIAH2 is an oncogene reportedly can affect the stability of HIF-1*α* [30]. It is reported that the expression of SIAH2 in breast cancer is higher than that in healthy breasts, and the high expression of SIAH2 is associated with a decrease in disease-free survival in breast cancer patients [31]. Consist with these researches, this study found that the expression of SIAH2 and HIF-1*α* was enhanced in breast cancer SP cells exposed to hypoxia, along with the increased EMT transformation and VM formation. It is reported that administration of sinomenine can reduce the serum levels of HIF-1*α* and inhibit angiogenesis in a mouse model of collagen-induced arthritis [32]. In this study, sinomenine was found to neutralize hypoxia-induced upregulation of SIAH2 and HIF-1*α*, as well as inhibit EMT transformation. Besides, overexpression of SIAH2 obviously alleviated the anti-EMT effect of sinomenine, along with upregulation of HIF-1*α*. Therefore, sinomenine can inhibit hypoxia-mediated EMT process of breast cancer SP cells by repressing the SIAH2/HIF-1*α* axis.

Multiple miRNAs, such as miR-324-5p and miR-340-5p [9, 25], were reported to be involved in cell carcinogenesis through regulation of multiple genes, including SIAH2 [33]. MiR-340-5p, which was downregulated in the more aggressive breast cancer cell lines [25], was confirmed to target and downregulate SIAH2 in this study. Hypoxia-caused activation of SIAH2/HIF-1*α* pathway in breast cancer SP cells was also repressed by miR-340-5p mimics. Through inhibiting the proliferative, invasive, and migrating capabilities of breast cancer cells, miR-340-5p overexpression can impede breast cancer progression by targeting and modulating multiple genes [25, 34, 35]. Our previous study found that sinomenine can inhibit breast cancer progression via regulating miR-324-5p [9]. This study found that sinomenine can upregulate miR-340-5p expression in breast cancer SP cells under hypoxic conditions. Moreover, both miR-340-5p inhibitor and SIAH2 overexpression partly counteracted the inhibitory effects of sinomenine on hypoxia-triggered VM formation and metastasis of breast cancer SP cells. These findings are similar to the studies showing that miR-340-5p knockdown and SIAH2 overexpression exert cancer-promoting effects in breast cancer [25, 36]. However, the regulation of sinomenine on miR-340-5p and SIAH2 needs to be validated in more cell lines and model animals in the future.

## 5. Conclusion

Taken together, sinomenine can inhibit hypoxia-triggered VM formation and metastasis of breast cancer SP cells by regulating the miR-340-5p/SIAH2 axis.

Sinomenine and miR-340-5p/SIAH2 axis may be the promising candidates for the treatment of breast cancer.

## Data Availability

All the data involved in this study have been included within the article.

## Abbreviations

CSCs: Cancer stem cells
EMT: Epithelial-mesenchymal transition
HIF-1*α*: Hypoxia inducible factor-1*α*
MMPs: Matrix metalloproteinases
miRs: microRNAs
SP: Side population
VE-cadherin: Vascular endothelial-cadherin
VM: Vasculogenic mimicry.

## Supplementary Materials

Supplementary Materials Figure S1: effects of sinomenine on the survival of breast cancer SP cells. Breast cancer SP cells were incubated with different doses (0, 0.25, 0.5, 0.75, and 1 mM) of sinomenine under normoxic or hypoxia for 24 hours, and cell viability was analyzed by CCK-8 assay. Data are expressed as the means ± standard deviation from 3 independent experiments.

## References

[1] Bray F., Ferlay J., Soerjomataram I., Siegel R. L., Torre L. A., Jemal A. (2018). Global cancer statistics 2018: GLOBOCAN estimates of incidence and mortality worldwide for 36 cancers in 185 countries. *CA: a Cancer Journal for Clinicians*.

[2] Zhang J., Qiao L., Liang N. (2016). Vasculogenic mimicry and tumor metastasis. *Journal of BUON*.

[3] Andonegui-Elguera M. A., Alfaro-Mora Y., Caceres-Gutierrez R., Caro-Sanchez C. H. S., Herrera L. A., Diaz-Chavez J. (2020). An overview of vasculogenic mimicry in breast cancer. *Frontiers in Oncology*.

[4] Fan Y. L., Zheng M., Tang Y. L., Liang X. H. (2013). A new perspective of vasculogenic mimicry: EMT and cancer stem cells (review). *Oncology Letters*.

[5] Britton K. M., Kirby J. A., Lennard T. W., Meeson A. P. (2011). Cancer stem cells and side population cells in breast cancer and metastasis. *Cancers (Basel)*.

[6] Wang M., Wang Y., Zhong J. (2015). Side population cells and drug resistance in breast cancer. *Molecular Medicine Reports*.

[7] Jin C., Zou T., Li J. (2015). Side population cell level in human breast cancer and factors related to disease-free survival. *Asian Pacific Journal of Cancer Prevention*.

[8] Song L., Liu D., Zhao Y. (2018). Sinomenine reduces growth and metastasis of breast cancer cells and improves the survival of tumor-bearing mice through suppressing the SHh pathway. *Biomedicine & Pharmacotherapy*.

[9] Song L., Liu D., Zhao Y. (2015). Sinomenine inhibits breast cancer cell invasion and migration by suppressing NF-*κ*B activation mediated by IL-4/miR-324-5p/CUEDC2 axis. *Biochemical and Biophysical Research Communications*.

[10] Gao L. N., Zhong B., Wang Y. (2019). Mechanism underlying antitumor effects of Sinomenine. *Chinese Journal of Integrative Medicine*.

[11] Song L., Zhang H., Hu M. (2021). Sinomenine inhibits hypoxia induced breast cancer side population cells metastasis by PI3K/Akt/mTOR pathway. *Bioorganic & Medicinal Chemistry*.

[12] Xie T., Ren H. Y., Lin H. Q. (2016). Sinomenine prevents metastasis of human osteosarcoma cells via S phase arrest and suppression of tumor-related neovascularization and osteolysis through the CXCR4-STAT3 pathway. *International Journal of Oncology*.

[13] Li X., Li P., Liu C. (2017). Sinomenine hydrochloride inhibits breast cancer metastasis by attenuating inflammation-related epithelial-mesenchymal transition and cancer stemness. *Oncotarget*.

[14] Zhang H., Ren Y., Tang X. (2015). Vascular normalization induced by sinomenine hydrochloride results in suppressed mammary tumor growth and metastasis. *Scientific Reports*.

[15] Godet I., Shin Y. J., Ju J. A., Ye I. C., Wang G., Gilkes D. M. (2019). Fate-mapping post-hypoxic tumor cells reveals a ROS-resistant phenotype that promotes metastasis. *Nature Communications*.

[16] Koh S. Y., Moon J. Y., Unno T., Cho S. K. (2019). Baicalein suppresses stem cell-like characteristics in radio- and chemoresistant MDA-MB-231 human breast cancer cells through up-regulation of IFIT2. *Nutrients*.

[17] Masoud G. N., Li W. (2015). HIF-1 *α* pathway: role, regulation and intervention for cancer therapy. *Acta Pharmaceutica Sinica B*.

[18] Vaupel P., Multhoff G. (2018). Hypoxia-/HIF-1*α*-driven factors of the tumor microenvironment impeding antitumor immune responses and promoting malignant progression. *Advances in Experimental Medicine and Biology*.

[19] Azad T., Janse van Rensburg H. J., Lightbody E. D. (2018). A LATS biosensor screen identifies VEGFR as a regulator of the hippo pathway in angiogenesis. *Nature Communications*.

[20] Wang M., Zhao X., Zhu D. (2017). HIF-1*α* promoted vasculogenic mimicry formation in hepatocellular carcinoma through LOXL2 up-regulation in hypoxic tumor microenvironment. *Journal of Experimental & Clinical Cancer Research*.

[21] Chan P., Moller A., Liu M. C. (2011). The expression of the ubiquitin ligase SIAH2 (seven in absentia homolog 2) is mediated through gene copy number in breast cancer and is associated with a basal-like phenotype and p53 expression. *Breast Cancer Research*.

[22] Qi J., Nakayama K., Gaitonde S. (2008). The ubiquitin ligase Siah2 regulates tumorigenesis and metastasis by HIF-dependent and -independent pathways. *Proceedings of the National Academy of Sciences of the United States of America*.

[23] Zhang X., Li Y., Ma Y. (2018). Yes-associated protein (YAP) binds to HIF-1*α* and sustains HIF-1*α* protein stability to promote hepatocellular carcinoma cell glycolysis under hypoxic stress. *Journal of Experimental & Clinical Cancer Research*.

[24] Ma B., Chen Y., Chen L. (2015). Hypoxia regulates hippo signalling through the SIAH2 ubiquitin E3 ligase. *Nature Cell Biology*.

[25] Wu Z. S., Wu Q., Wang C. Q. (2011). miR-340 inhibition of breast cancer cell migration and invasion through targeting of oncoprotein c-met. *Cancer*.

[26] Li S., Meng W., Guan Z., Guo Y., Han X. (2016). The hypoxia-related signaling pathways of vasculogenic mimicry in tumor treatment. *Biomedicine & Pharmacotherapy*.

[27] Wang H. J., Guo Y. Q., Tan G. (2013). miR-125b regulates side population in breast cancer and confers a chemoresistant phenotype. *Journal of Cellular Biochemistry*.

[28] Jiang Y., Jiao Y., Liu Y. (2018). Sinomenine hydrochloride inhibits the metastasis of human glioblastoma cells by suppressing the expression of matrix Metalloproteinase-2/-9 and reversing the endogenous and exogenous epithelial-mesenchymal transition. *International Journal of Molecular Sciences*.

[29] De Francesco E. M., Maggiolini M., Musti A. M. (2018). Crosstalk between notch, HIF-1*α* and GPER in breast cancer EMT. *International Journal of Molecular Sciences*.

[30] Xu D., Li C. (2021). Regulation of the SIAH2-HIF-1 Axis by protein kinases and its implication in cancer therapy. *Frontiers in Cell and Development Biology*.

[31] Knauer S. K., Mahendrarajah N., Roos W. P., Kramer O. H. (2015). The inducible E3 ubiquitin ligases SIAH1 and SIAH2 perform critical roles in breast and prostate cancers. *Cytokine & Growth Factor Reviews*.

[32] Feng Z. T., Yang T., Hou X. Q. (2019). Sinomenine mitigates collagen-induced arthritis mice by inhibiting angiogenesis. *Biomedicine & Pharmacotherapy*.

[33] Kim Y., Kim H., Park D., Jeoung D. (2015). miR-335 targets SIAH2 and confers sensitivity to anti-cancer drugs by increasing the expression of HDAC3. *Molecules and Cells*.

[34] Meng L., Yue X., Zhou D., Li H. (2020). Long non coding RNA OIP5‑AS1 promotes metastasis of breast cancer via miR‑340‑5p/ZEB2 axis. *Oncology Reports*.

[35] Yu Y., He Y., Shao Y., Chen Q., Liu H. (2020). lncRNA PCNAP1 predicts poor prognosis in breast cancer and promotes cancer metastasis via miR‑340‑5p‑dependent upregulation of SOX4. *Oncology Reports*.

[36] Sun J., Zhang X., Han Y., Zhen J., Meng Y., Song M. (2016). Overexpression of seven in absentia homolog 2 protein in human breast cancer tissues is associated with the promotion of tumor cell malignant behavior in in vitro. *Oncology Reports*.

