# Supplementary material for: Sinomenine Inhibits Vasculogenic Mimicry and Migration of Breast Cancer Side Population Cells via Regulating miR-340-5p/SIAH2 Axis
Source: Biomed Res Int. 2022 Mar 9;2022:4914005. doi: 10.1155/2022/4914005 (PMC8926463; doi:10.1155/2022/4914005)
Supplement: Supplementary Materials — Figure S1: effects of sinomenine on the survival of breast cancer SP cells. Breast cancer SP cells were incubated with different doses (0, 0.25, 0.5, 0.75, and 1 mM) of sinomenine under normoxic or hypoxia for 24 hours, and cell viability was analyzed by CCK-8 assay. Data are expressed as the means ± standard deviation from 3 independent experiments. [file 4914005.f1.docx]

**Supplementary materials**

**Figure S1**


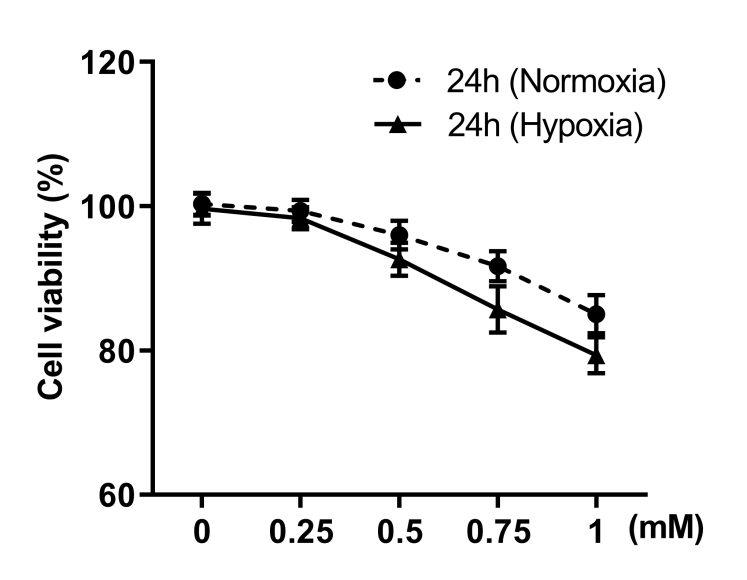


**Figure S1 Effects of sinomenine on the survival of Breast cancer SP cells.**

Breast cancer SP cells were incubated with different doses (0, 0.25, 0.5, 0.75 and 1 mM) of sinomenine under normoxic or hypoxia for 24 hours, and cell viability was analyzed by CCK-8 assay. Data are expressed as the mean ± standard deviation from 3 independent experiments.
